# Simulated fire video collection for advancing understanding of human behavior in building fires

**DOI:** 10.3389/fpsyg.2024.1438020

**Published:** 2024-08-26

**Authors:** Justin W. Bonny, Zishanul Hussain, Micah D. Russell, Arnaud Trouvé, James A. Milke

**Affiliations:** ^1^Department of Psychology, Morgan State University, Baltimore, MD, United States; ^2^Department of Fire Protection Engineering, University of Maryland, College Park, College Park, MD, United States

**Keywords:** fire safety, risk perception, flames, smoke, egress, human behavior in fire

## Abstract

**Introduction:**

The goal of the present research was to develop a video collection of simulated fires to investigate how people perceive growing building fires. In fire safety science, a critical factor to occupant responses to building fires is the pre-movement period, determined by how long it takes an individual to initiate taking protective action with an incipient fire. Key to studying the psychological processes that contribute to the duration of the pre-movement period is presenting human subjects with building fires. One approach used in previous research is to present videos of building fires to individuals via scenarios. The numerical simulations used to model fire dynamics can be used to render videos for these scenarios. However, such simulations have predominantly been used in fire protection engineering to design buildings and are relatively inaccessible to social scientists.

**Method:**

The present study documents a collection of videos, based on numerical simulations, which can be used by researchers to study human behavior in fire. These videos display developing fires in different types of rooms, growing at different rates, different smoke thickness, among other characteristics. As part of a validation study, participants were presented with subsets of the video clips and were asked to rate the perceived risk posed by the simulated fire.

**Results and discussion:**

We observed that ratings varied by the intensity and growth rate of the fires, smoke opacity, type of room, and where the viewpoint was located from the fire. These effects aligned with those observed in previous fire science research, providing evidence that the videos could elicit perceived risk using fire simulations. The present research indicates that future studies can utilize the video library of fire simulations to study human perceptions of developing building fires as situational factors are systematically manipulated.

## Introduction

Understanding human behavior in fire (HBIF) is crucial to limiting casualties in residential fire situations. Within the United States, around 350,000 residential fires are reported each year with nearly 3,000 people killed in fire incidents (U.S. Fire Administration, 2024). There remain, however, two main challenges to studying human behavior in residential fires. First, prior research provides evidence that human perceptions and action responses are multiply determined, both by characteristics of the fire (e.g., smoke thickness) and the environment (e.g., location of fire within building; e.g., Bryan, 1977). Second, real fire events pose a number of risks to bystanders making them impractical in human subjects research settings. These challenges, in part, can be addressed using fire simulations as part of laboratory experiments. Simulations offer the opportunity for precise control over fire and building characteristics via numerical models that are used in fire protection engineering research (McGrattan et al., 2021). However, the expertise and computational requirements of such programs pose a barrier to the use of numerical simulations of fire by researchers in interdisciplinary fields, including the social sciences. To address this, the present research introduces and evaluates an array of videos of simulated incipient residential fires for use by interdisciplinary researchers to study human behavior in fire.

### Contributing factors to occupant responses to building fires

A crucial component to building life safety design is ensuring that occupants have enough time to make it to safety when an emergency occurs. In fire protection engineering, the required safe egress time (RSET) is the estimated amount of time required for occupants to move to a safe location after a fire is ignited within a building (Society of Fire Protection Engineers, 2019a). An accurate estimate of RSET is essential for incorporating design elements into buildings to provide an available safe egress time (ASET) with a sufficient safety margin for building occupants (Hurley et al., 2015). Several behavioral models have been developed to capture the different mental and physical processes that contribute to RSET (Kuligowski, 2015). The Protective Action Decision Model (PADM), originally developed to account for human responses to emergencies and disasters (Lindell and Perry, 2012), has been applied to HBIF (Kuligowski, 2013). The pre-movement period, which begins when an occupant receives fire cues and ends when a protective action is initiated (Society of Fire Protection Engineers, 2019b), contains several key phases of PADM that contribute to how quickly occupants begin taking protective action (Kuligowski, 2009). During Phase 1, occupants need to detect, pay attention to, and comprehend, relevant perceptual cues (e.g., visible smoke, alarm sound) as indicative of a fire emergency. In Phase 2 of PADM, the occupant uses the detected cues to identify and interpret the posed risk of the situation. This culminates in Phase 3 where the occupant decides whether and what protective action to take, initiating and performing the action in Phase 4. With regard to estimating RSET during incipient fires, delays in Phases 1 and 2 of PADM from ambiguity, misinterpretation, or cognitive biases can have a cascading effect on when occupants begin taking protective action (Fischhoff and MacGregor, 1982; Kuligowski and Mileti, 2009). Identifying what factors influence the perception and interpretation of fire cues during these early phases can contribute to more accurate estimates of RSET, maximizing the chance that ASET is sufficiently planned for building life safety systems.

### Impact of fire and environmental factors on perceived risk

Prior HBIF research has identified how connections between human responses and fire characteristics can be investigated using a continuum of data sources ranging from real-life incidents to simulated lab experiments. Across this continuum, evidence that fire characteristics influence human responses has come from multiple research methods, including case study interviews with survivors of fire incidents as well as laboratory experiments with the general public (Sime, 1984; Tong and Canter, 1985; Kinateder et al., 2014; Kuligowski, 2017). In studying human behavior during building fires, the data with the highest ecological validity can be gathered from field-based research such as post fire incident surveys (Kinateder et al., 2014). The factors observed to correlate with human behavior in such field research can then be further investigated with laboratory experiments that have lower ecological validity. With regard to fire incident studies, Bryan (1977) completed post-incident interviews with occupants that experienced a fire emergency, focusing on the characteristics and actions that they performed. Across incidents, occupants reported attending to multiple perceptual characteristics of the fire during the emergency, including the thickness and smell of smoke, sight of flames when encountered, and sounds of alert systems (Bryan, 1977). Furthermore, environmental factors, specifically the room and location that contained the fire relative to the occupant, had an impact on the sequence of actions that occupants took (Bryan, 1977). These results, as well as other post-incident studies (Sime, 1984; Tong and Canter, 1985), indicate that occupants during a building fire detect and perceive several characteristics which influenced their behavior during the incident.

Post fire incident data has been used to identify contributing factors to risk perception during fire events. A review by Kinateder et al. (2015) focused on risk perception of fires during building evacuations as reported across several types of field-research data collection methods, including questionnaires and interviews, to identify common trends. As part of the review, the authors provided a definition of risk perception specific to occupants facing a fire, “… the perception of an imminent threat to one’s own life and health” (Kinateder et al., 2015, p. 6). Using this definition as guidance, across studies, higher ratings and reports of risk perception were more likely to occur with fires when occupants reported encountering more cues, such as both flames and smoke, and when the location of the occupant was on higher building levels (Kinateder et al., 2015). This similar pattern across different types of data collection methods indicates that characteristics of fires and the environment influence the perceptions and actions of occupants. Although post-incident reports are limited due to survivorship bias, where responses are not available from those that perished (Savage and Torgler, 2021), corroborating evidence has been gathered from fire scenarios presented as part of lab-based experiments.

Using effects observed in field-based research as a starting point, laboratory experiments have been used to further investigate factors that influence the perceived risk of building fires. During fire scenario research, participants are presented with hypothetical situations that include a fire and are asked to make judgments about it. The experimental control over the hypothetical situations allows researchers to manipulate factors related to the fire and environment, which are not feasible with real-life fire events, and assess the impact on participant behaviors (Kinateder et al., 2014). It is important to note, however, that the operationalization of risk perception in hypothetical studies varies from that provided by Kinateder et al. (2015) for real-life fires. As noted in prior research, hypothetical studies tend to lack the ecological validity of real fire incidents (Kinateder et al., 2014). In addition, hypothetical studies are unable to approach the chance of personal harm as real fire emergencies pose to occupants. Instead, hypothetical studies of fire events are more closely aligned with hazardous events that can occur in the environment, including natural and human-made disasters (Slovic, 1987). In this manner, the perceived risk of hypothetical fire events corresponds to the potential threat posed by a hazard in the environment (Kraus et al., 1992; Slovic and Weber, 2002). Using such an approach has revealed similar patterns as interview and questionnaire studies conducted after fire incidents. Scenarios that presented videos of real fires which contained larger flames and amounts of smoke tended to be judged as more dangerous (Bonny and Leventon, 2021) and as requiring actions to disengage with the hazard (Hulse et al., 2020). Simulated fires have also been used in hypothetical studies with, compared to videos of real fires, the advantage of being able to be used with building environments where real fires have not been filmed and disadvantage of fires visually appearing less realistic. For example, thicker smoke emitted from simulated fires has been observed to influence the evacuation route selection of participants completing a virtual reality-based task (Fu et al., 2021). Although the number of fire characteristics that have been investigated across these methods is limited, the available evidence suggests that, to an extent, those observed to have an impact in real-life fires can also impact behavior in hypothetical studies that use videos of real and simulated fires.

There is a lack of research investigating the impact of environmental factors observed with post incident interview and questionnaire studies, including room type and proximity to fire, on the perceived risk of fires during laboratory-based scenarios. In line with PADM, the environment (including physical and social aspects) plays an important role in peoples’ understanding of perceived risk (Lindell and Perry, 2012). Furthermore, other characteristics of buildings, including escape route signage and familiarity with the building have been observed to impact occupant behaviors during fire events (Kobes et al., 2010). Investigating the impact of environmental characteristics on the perceived risk of fire scenarios in connection with fire characteristics can provide greater insight into how these factors combine to influence human perceptions of fire.

### Stimuli based on simulations of building fires

Numerical simulations provide a method for generating visual renderings of building fires for scenario-based research. Computational models have been implemented in software packages to simulate the fluid dynamics of fire for use in fire science research as well as performance-based approaches for building safety design (Gernay, 2024). Although there are limitations, validation studies evaluating the software have observed that numerical simulations can be used to estimate the behavior of real fires (Yuen et al., 2014). For example, the time progression of fires can be simulated using different intensities, growth rates, combustion properties, and smoke production within different building plans and room geometries. Indeed, government agencies in some jurisdictions require the use of numerical simulations to demonstrate that planned buildings provide sufficient ASET for future occupants to take protective action during fire events (e.g., MBIE, 2014). From the perspective of HBIF research, simulations afford the ability to model fires while systematically varying fire and environment characteristics. Combined with visual renderings, fire and environment characteristics observed in prior research to be connected with human behaviors and risk perception can be systematically manipulated. A prevalent simulation software, Fire Dynamics Simulator (FDS; McGrattan et al., 2021), has such capabilities when combined with rendering programs. Developed by the National Institute of Standards and Technology (NIST), it is an eddy based computational fluid dynamics model of fire-driven flow that uses formula driven software with a focus on smoke and heat rates of fire to simulate movement (McGrattan et al., 2021). In addition to Smokeview (Forney, 2023), PyroSim (PyroSim User Manual, 2023) can render FDS computational output as three-dimensional visualizations, including flames and smoke; PyroSim has the additional capability of rendering furnished rooms created in computer aided design (CAD) software. A drawback is that, although the rendered fires can perform like those in the real world, the visualizations look similar to real fires but are lacking in realism. Nonetheless, the benefits of computationally-derived fires have led to them being incrementally used in HBIF research fire scenario studies. For example, FDS-based renderings have been used with human participants in laboratory research to examine the visual perception of fire intensity (Bonny and Milke, 2023) and building evacuation planning (Yan et al., 2017, 2020). In the absence of practical methods of exposing human participants to real building fires, renderings of simulated fires offer an approach for using experimental designs to study HBIF.

A barrier to the wider use of fire simulations in HBIF research is the technical complexity of the software and the coding therein. The technical requirements of creating and running the simulation software include knowledge of combustion and building systems design as well as the computational hardware for running the numerical models. This has, in part, contributed to fire simulations predominantly being used by fire scientists and fire protection engineers. To expand and test models of HBIF, such as PADM, specifically as to how fire and environment characteristics combine to influence perceptions of risk, necessitates interdisciplinary research with social scientists. An approach for doing so is to create a preexisting resource of visually rendered simulations using fire modeling software so that researchers can use these in HBIF research without necessitating a high-level of technical knowledge and computational resources.

### The present study

The goals of the present study were to create a repository of video renderings of fire simulations and to validate these videos for use in hypothetical scenario-based human behavior in fire research. To do so, we created numerical simulations of incipient fires (by means of FDS) and, using PyroSim, rendered these simulations as videos. Using these two software packages, a collection of videos was created to systematically manipulate the following fire and environment characteristics: growth rate (how quickly fires increased in heat release rate), intensity of the fire (heat release rate, based on how long the fire had been growing), smoke opacity (how visually transparent smoke appeared), type of residential rooms (bedroom, living room, kitchen, office), and viewpoint distance (how far the video camera was from the fire within the virtual room). To evaluate the extent to which the videos elicited perceived risk, participants rated a set of clips from the videos. In the present study, we define perceived risk as the judged harmful potential of a fire. Using this definition, we investigated whether manipulating fire and environment characteristics elicited different levels of perceived risk, similar to prior HBIF research findings. To do so, we presented portions of the videos and had participants rate the level of danger posed within the video clips.

The methods used in the present study were motivated by prior research approaches that used media-based stimuli to evoke emotion responses. Past studies have presented evocative visual stimuli to participants while collecting ratings of emotion valence and arousal; this includes pictures (e.g., International Affective Picture System, IAPS, Lang et al., 2008) and computer-generated videos (Courtney et al., 2010). The collected measures were used to evaluate the affective judgments of individuals based on the representations evoked by stimuli. Although these studies used media-based stimuli, researchers have posited that the information contained within them, such as pictures, can, at least partially, activate representations of the corresponding real-life objects and associated emotional responses (Lang et al., 1993). Indeed, studies have provided evidence that systematically varying pictures and videos that differed in the arousal and valence displayed were effective in eliciting emotion responses as measured by participant emotion ratings and psychophysiological responses (Bradley and Lang, 1994; Cuthbert et al., 2000; Courtney et al., 2010). As applied to the present research, if simulated fire videos were perceived as representing real fires, we hypothesized that the perceived potential danger of the fires would align with observations made in prior HBIF research. We predicted higher perceived risk ratings would be observed for fires that grew faster, fires with thicker smoke, higher intensity fires, for rooms where fires were believed to be more likely to occur, and viewpoints that were closer to the fire in the simulated room.

## Methods

The present research had two main components: the generation of a video library based on the numerical simulations of developing fires and behavioral data collection to evaluate the video library for use in fire risk perception research. The two components are described in the respective order with additional information contained within Supplementary materials.

### Simulated fire growth

The present series of simulations consider fires featuring multiple growth rates. Combustion properties were specified using the FDS syntax. FDS is a computational model that numerically simulates combustion using large eddy simulation (LES) models (McGrattan et al., 2021). Polyurethane (GM27; Hurley et al., 2015) was utilized for the chemical reaction (combustion properties are available in Supplementary materials). In the conducted simulations, six distinct *t*-squared growth rate fires were analyzed: slow, medium, quick, fast, ultra-fast, and warp. Apart from quick and warp, the growth rates aligned with those used in fire safety science (Kim and Lilley, 2002). A *t*-squared growth rate is represented by the equation $Q=\alpha t^{2}={\left(t/t_{g}\right)}^{2}\times 1055$ (kW) where *t* represents time in seconds, and *t_g_* denotes the time in seconds to reach a heat release rate (HRR) of 1,055 kW (1,000 Btu/s, the time required to reach 1,055 kW is shown in Table 1; Ciani and Capobelli, 2018). In addition to quantifying the intensity of the energy output of the fire, HRR provides a measure of the hazard associated with the fire that is posed to occupants (Babrauskas and Peacock, 1992).

**Table 1:** Fire growth rates used across simulations.

Growth rate	$t_{g}$ (kW)^1^	ɑ^1^	Radius per second (m)
Slow	600	0.00293	0.000483
Medium	300	0.01172	0.000966
Quick	204	0.0253	0.00142
Fast	150	0.0469	0.00193
Ultra	75	0.1876	0.00386
Warp	40	0.6954	0.00725

^1^ $t_{g}$ = time to reach 1.055 MW; ɑ = alpha coefficient in t-squared equation that corresponds to growth rate.

In the present configuration, the fire burner measured 0.5 m by 0.5 m. The fire originated at the center of the burner and spread outwards in a radial pattern. The velocity of this radial spread depended on the fire growth rate. The radial spread rates, as entered into the FDS, were derived from the growth rates presented in Table 1. The slow growth rate resulted in a radial spread rate of 0.000483 m/s, while the warp growth rate, which was the fastest, had a radial spread rate of 0.00725 m/s.

Two software options considered for visually rendering the output of FDS simulations for the present research were Smokeview (NIST) and PyroSim (Thunderhead). Smokeview was the default visualization tool for outputs from FDS simulations; FDS, by default generates Smokeview files for visual inspection. Although capable of displaying 3D objects and various types of output data, Smokeview uses the rectangular Cartesian computational mesh system used by FDS and lacked support for rendering realistic 3D geometries. Typically, a realistic 3D object in FDS is fragmented into distinguishable cubes, resulting in an unrealistic appearance. PyroSim software (Thunderhead Engineering Consultants, Inc.) offered the capability to import virtual 3D objects, such as furniture and building models, into an FDS computational domain. Internally, it generates an FDS input code that decomposes the 3D object into small solid obstructions based on the mesh size. When viewing FDS and Smokeview output in PyroSim, the 3D objects are reassembled as they were originally input, making them appear significantly more realistic compared to those displayed in Smokeview. The input files for each room type, incorporating various pieces of furniture, were created by PyroSim and executed independently in FDS, with the results subsequently visualized in PyroSim. For the present research, 3D furniture models were sourced from https://www.turbosquid.com/.

**Figure 1:**
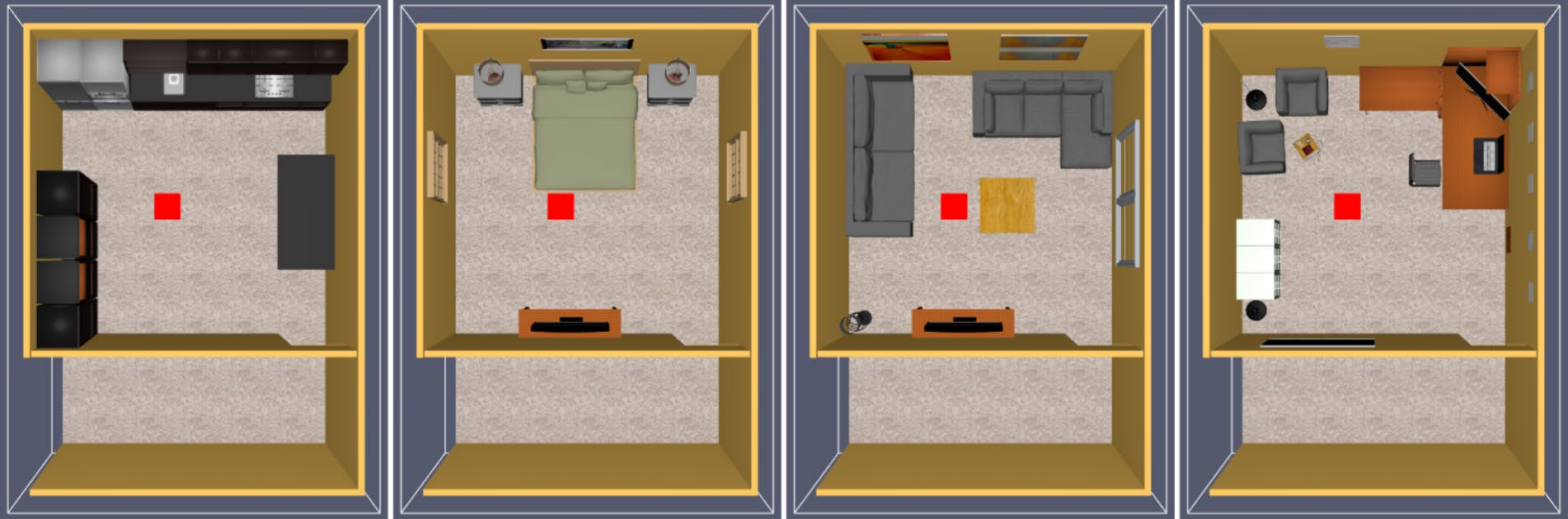
Perspective view of the three-dimensional rooms constructed for the present research (from left to right: kitchen, bedroom, living room, office). The red square near the center of the room was the location of the fire.

In the framework of the present study, four distinct types of rooms were considered - a bedroom, a kitchen, a living room, and an office - utilizing various types of 3D objects that were representative of each environment (see Figure 1). For instance, the bedroom simulation incorporated elements such as a king-sized bed and side tables, while the kitchen was outfitted with cabinets, a refrigerator, and a gas stove, among other items. For each type of room, six simulations were performed corresponding to the six values of the selected growth rates (slow, medium, quick, fast, ultra, warp; see Table 1). The volume of the room was 5 m by 5 m by 2.8 m; the configuration included a 2-m-wide hallway, and featured a door measuring 0.91 m by 2.13 m.

### Numerical fire simulations

For computational analysis, the spatial regions of each room were divided into computational cells that represented the entirety of the spatial region under study. The cell size denotes the discrete subdivisions of this domain, determining the resolution at which numerical simulations of phenomena—such as flow, combustion, heat and mass transfers—are examined and modeled in FDS. For the present study, the size of the computational domain was 5.5 m by 7.5 m by 3.5 m (height). A cell size of 2.5 cm was selected to strike a balance between computational cost and the expected level of realism in the visualizations of simulated fire and smoke. This choice resulted in a total of 9.24 million cells, which were subsequently divided into 10 separate meshes. Each mesh was processed in parallel, utilizing Message Passing Interface (MPI) protocols for distributed computation on the University of Maryland’s (UMD) Zaratan High-Performance Computing (HPC) cluster.

Two test simulations were conducted to evaluate the visual fidelity of simulated fire and smoke depictions in video playbacks: one at 30 frames per second and the other at 60 frames per second. The simulation parameter of 60 frames per second provided a more life-like representation and was selected for the final simulations. For a single setup, the simulation required 1,369 s, as detailed in Table 1. To conduct simulations across four distinct room configurations, a cumulative 5,476 s was necessary. In total, the completion of all simulations consumed 28,783 CPU hours.

### Video library of fire simulations

Videos were generated for each fire simulation using the PyroSim software. Along with numerical estimates of combustion behavior from FDS, Smokeview estimates the visual appearance of the fire using the radiation transport equation (Forney, 2023). By default, FDS simulations generate Smokeview files for visually rendering simulation output. These output files can be rendered by both Smokeview and PyroSim; PyroSim uses these estimates to visually render the luminosity and opacity of flames and smoke (PyroSim User Manual, 2023). These visualizations can be rendered from specific viewpoints by placing cameras at specific locations and orientations that correspond to the perspective of a simulated human observer. In the present research, each numerical simulation was rendered from several viewpoints (camera placed at a height of 1.62 m at each viewpoint) as an MP4 video (540 by 960 pixels, 60 frames per second) for the entire duration of the simulation. The video library collection is available online via a FigShare repository (Bonny, 2024a). Short clips from these videos were used to evaluate whether the videos were perceived as displaying room fires that posed risk.

### Materials

All behavioral tasks were coded using the jsPsych java script library (de Leeuw, 2015) and hosted online. Participants were able to complete the study using an internet-connected device with tasks programmed to accept touchscreen and mouse along with keyboard input. Videos were hosted using the Vimeo platform (Vimeo.com, Inc.) and were streamed to participant devices during the tasks. A JATOS server (Lange et al., 2015) hosted on the DigitalOcean platform (DigitalOcean Holdings, Inc.) was used to manage study files and collect participant data.

For the study, short segments from simulation library videos were presented to participants as video clips. Each clip was 8 s in duration and rendered to be viewable on both computer and smartphone screens (360 by 630 pixels, 30 frames per second). The starting times of video clips were selected to display developing fires at different levels of intensity. Specifically, the start time of video clips was calculated as the time at which the subsequent 8 s of the simulation had a mean HRR of the specified intensity (in kW: 108, 277, 446, 615).

### Participants

A total of 2,158 participants, recruited from the online participant panel Prolific, completed the study. Participants varied in age (*M* = 37.16, *SD* = 12.29, *Min* = 18, *Max* = 83), biological sex (*N* Female = 1,084, *N* Male = 1,074), and race (*N* White = 1745, *N* Asian = 164, *N* Black or African American = 132, *N* American Indian or Alaska Native = 17, *N* Native Hawaiian or Other Pacific Islander = 6, *N* multiple = 94). Eligibility was determined by participants responding that they were located within the United States, fluent in English, and had normal or corrected-to-normal vision. Participants received a monetary incentive of $1.25 (approximately 5 min to complete study). Participants provided informed consent with the study protocol in accordance with the Declaration of Helsinki and approved by the Institutional Review Board of Morgan State University (#22/05-0107).

### Experiment design

A mixed factorial design was used to collect ratings for all video clip stimuli. Across participants, video clips were varied by manipulating the type of room (four-levels; bedroom, living room, kitchen, office), viewpoint distance from fire (with the position varying from closer to the fire to closer to the room entrance; three-levels, in meters; 2.39, 3.39, 4.34), smoke opacity (three-levels, in percent of opaqueness with lower values corresponding to more transparent smoke: 0, 5, 10), and fire growth rate (six-levels; slow, medium, quick, fast, ultra, warp). Participants were randomly assigned to a between-subjects condition defined by these factors (a minimum number of 10 participants completed each condition). A within-subjects factor of fire intensity (in mean HRR during video clip; four-levels: 108, 277, 446, 615) was included, with each participant viewing fires of different intensities (random order). A single video clip was generated for each of the 864 conditions defined by the between- and within-subjects factors.

### Procedure

After viewing a brief description of the study posted to Prolific, eligible participants could begin the study by following the study URL via an internet browser. Participants were next presented with an informed consent form which indicated that they would be asked to view videos of simulated building fires. Those that consented were then presented with study instructions. They were informed that they would view several videos of building fires and be asked to rate each video as if they were a person who encountered the fire within a home. They were then presented with the experiment trials. For each trial, participants first selected a ‘start’ button to play the video and afterwards made three scale ratings about the video: “What was the severity of the fire” (1- very low, 9- very high), “There was risk of serious harm from the fire.” (1- strongly disagree, 9- strongly agree), “The fire posed imminent danger.” (1- strongly disagree, 9- strongly agree). The rating statements were selected to align with the operational definition of perceived risk within the present study and based on pilot data that indicated higher and lower ratings varied with corresponding changes in fire intensity. After making the ratings, the trial was completed, and participants were presented with the next trial. After completing all trials, participants were asked to self-report demographics and presented with a debriefing.

### Data preparation

An issue inherent in the use of simulated fires with different smoke opacities and growth rates was the accumulation of thick smoke that occluded the rooms for some of the video clips. This yielded a subset of trials where participants only viewed a dark grey screen for the duration of the video clip. These trials were identified as those where greater than 75% of the pixels in the frames of the first second of the video were dark grey (red, green blue, RGB, values less than 20, 20, 20; *N* dark trials = 5). For these trials, participants displayed greater variability in their responses (dark trial variance = 6.78; other trial variance = 4.33). Indeed, a Levene’s test comparing rating variance was significant, *F*(1, 26,632) = 35.21, *p* < 0.001, suggesting that there was greater ambiguity in how participants rated video clips for dark trials. To minimize the impact of such trials when analyzing the impact of factors, ratings for these trials were removed from further analysis. The dataset used for analyses is available via an online repository (Bonny, 2024b).

## Results

Although participants used a 1 through 9 scale to rate the videos, the numeric codes for the ratings ranged from 0 (i.e., rating of 1) through 8 (i.e., rating of 9). Data were analyzed using R (packages used: lme4, Bates et al., 2015; lmerTest, Kuznetsova et al., 2017; car, Fox and Weisberg, 2019; emmeans, Lenth et al., 2022; ggplot, Wickham, 2016) and tests were two-tailed (*α* = 0.05). Continuous factors were scaled and centered when entered as predictors and degrees of freedom for linear mixed models were estimated via the Satterthwaite method; *post hoc* comparisons were adjusted using Sidak corrections.

### Risk ratings analysis

Linear mixed regression models (random intercept for participant) were used to predict risk ratings. A stepwise approach was used to identify the best performing model when adding interaction effects. A forward selection process was used to add interaction effects between factors until a significant reduction in Bayesian Information Criterion (*BIC*) was no longer observed (Stoica and Selen, 2004). The baseline model included main effects of all factors; subsequent models added interaction effects starting with fire characteristics (baseline: *BIC* = 93,166; model 1: growth rate by intensity, *BIC* = 92,938; model 2: growth rate by intensity by smoke, *BIC* = 91,711) and then environment characteristics (model 3: growth rate by intensity by smoke by view distance, *BIC* = 91,748; model 4: growth rate by intensity by smoke by view distance by room type, *BIC* = 92,093). Using this process, model 2 was selected and used in subsequent analyses (baseline vs. model 1: Δ*BIC* = −278, *χ^2^* [1] = 237.5, *p* < 0.001; model 1 vs. model 2: Δ*BIC* = −1,227, *χ^2^* [3] = 1,258, *p* < 0.001; model 2 vs. model 3: Δ*BIC* = 37, *χ^2^* [7] = 33.96, *p* < 0.001).

**Figure 2:**
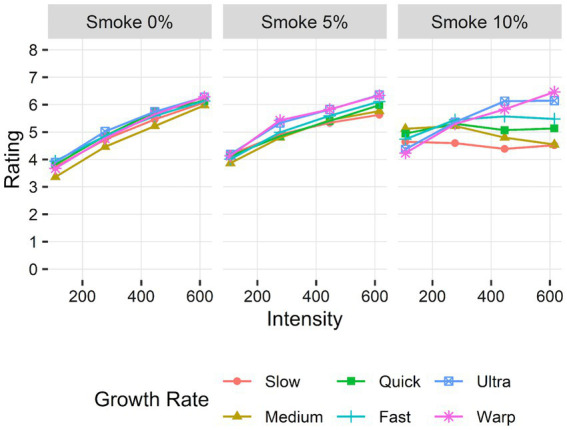
Mean risk ratings by fire intensity (heat release rate, in kW), growth rate, and smoke opacity.

For the selected model, a significant three-way interaction between growth rate, intensity, and smoke opacity was observed, *F*(1.00, 23738.00) = 210.42, *p* < 0.001, Cohen’s *f* = 0.09. Additional significant effects for fire characteristics were observed: main effects for growth rate and intensity, as well as interaction effects for growth rate by intensity, growth rate by opacity, and opacity by intensity (*p*s < 0.05; see Supplementary materials for statistics for each effect). *Post hoc* comparisons indicated that the main effects of fire characteristics, with higher ratings for more intense, opaque smoke, and faster growth fires, were moderated by a three-way interaction: the extent to which risk ratings increased with fire intensity and faster growth rates was affected by smoke opacity. Specifically, ratings were less likely to increase with intensity for slower growth rates as smoke opacity increased (Figure 2). Indeed, the slope between ratings and intensity significantly decreased as smoke opacity increased for all growth rates except for warp (adj. *p*s < 0.001).

**Figure 3:**
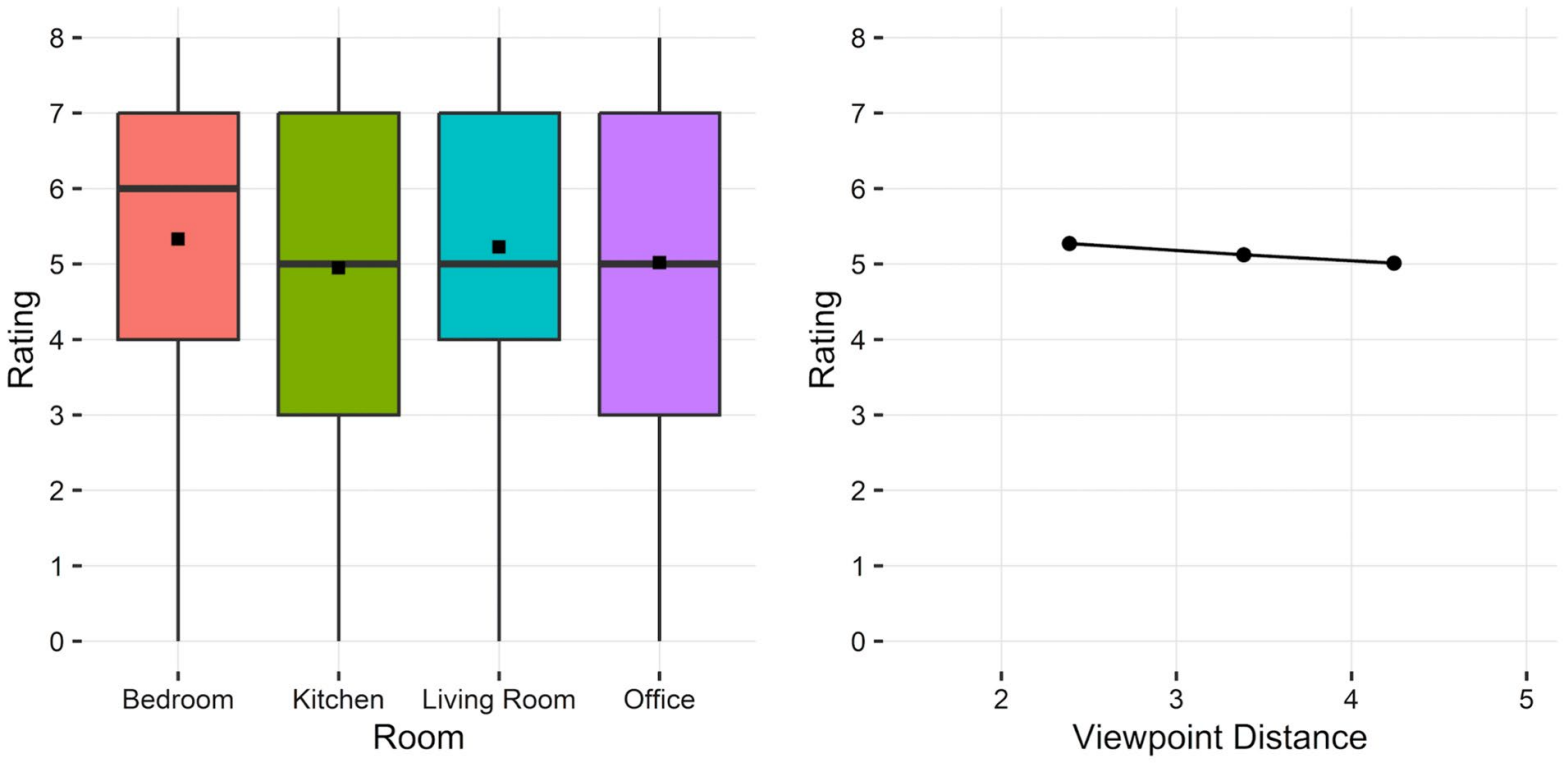
Distribution of risk ratings across room types (**left**; horizontal bars indicate median rating, dots indicate mean rating) and mean risk ratings across viewpoint distance (**right**; virtual distance from fire, in meters) conditions.

Significant main effects were observed for each of the environment variables. For view distance, participants provided higher risk ratings the closer the viewpoint was to the fire, *F*(1.00, 2158.00) = 10.19, *p* = 0.001, Cohen’s *f* = 0.07 (Figure 3). The significant effect of room type, *F*(3.00, 2158.00) = 7.38, *p* < 0.001, Cohen’s *f* = 0.10, was driven by higher ratings for the bedroom compared to kitchen and office conditions (adj. *p*s < 0.01) and higher ratings for the living room compared to kitchen conditions (adj. *p* = 0.015; all other *p*s > 0.1; Figure 3; see Supplementary materials for statistics for each effect).

### *Post hoc* experiment: viewpoint distance effect

The significant effect of viewpoint distance from the fire was further examined in a *post hoc* experiment. Two competing accounts for the observed effect were compared. In line with theories of embodied cognition (Wilson, 2002), the effect may have been due to participants simulating themselves within the room at a specific distance from the fire: standing further from the entrance, and closer to the fire, would be deemed riskier. Alternatively, the effect may have been due to the fire cues being physically larger on the screen for videos that were rendered from a closer viewpoint, regardless of the distance to the room entrance. This would be in line with prior observations that larger visual cues were associated with greater perceived risk of a room fire (Hulse et al., 2020). To compare these accounts, a new set of videos were rendered from two viewpoints that were equidistant from the fire but varied in egress path distance from the room entrance. This manipulation would further delineate the two accounts by incorporating the room entrance within the line of sight: since mental imagery may simulate the fire as larger when standing closer, incorporating the travel distance to the door would distinguish the prediction compared to the screen-size account. In line with the embodiment account, we predicted that participants would provide greater risk ratings when the viewpoint was from a position that would require farther travel to exit the room.

**Figure 4:**
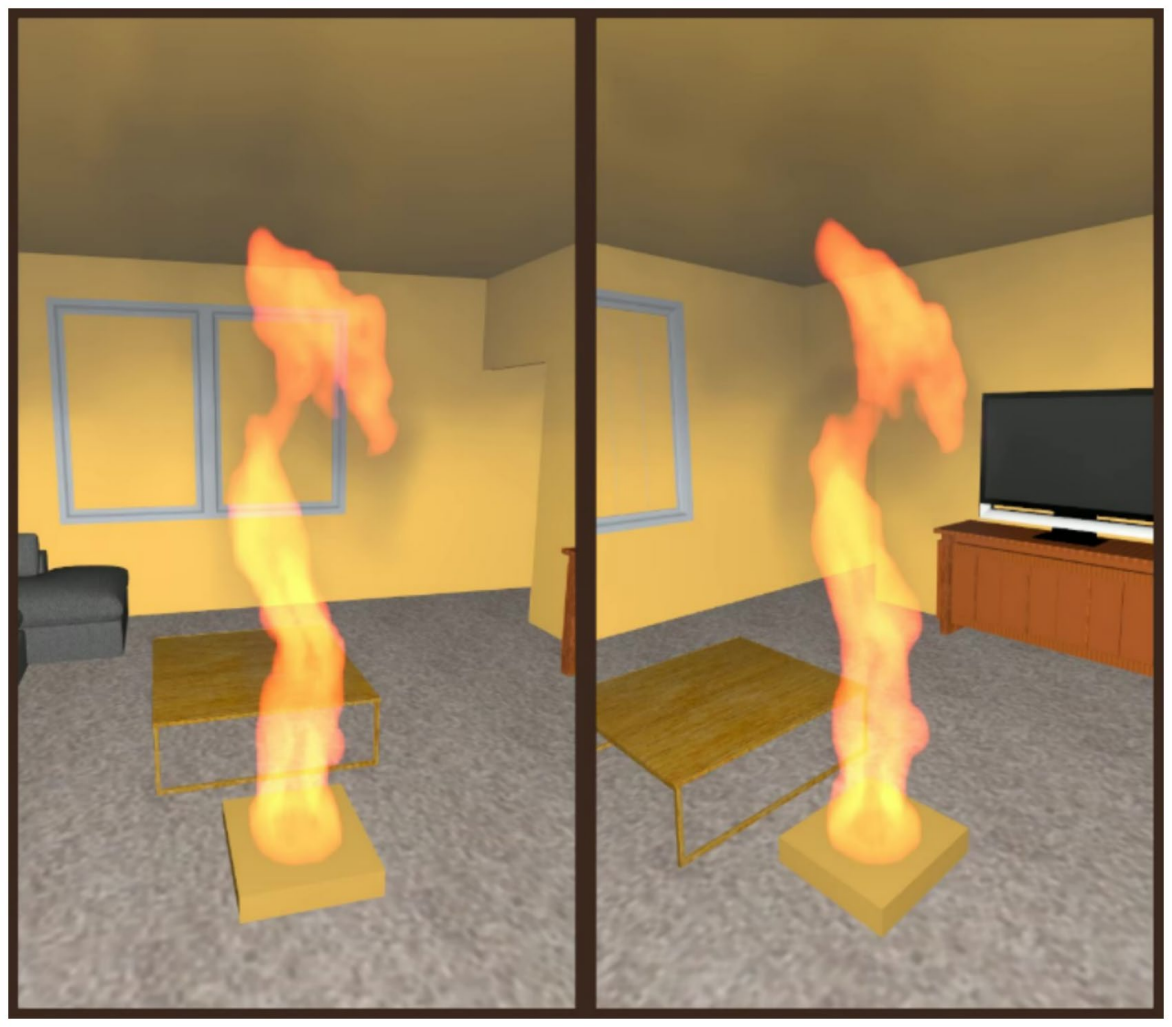
Still frames from the viewpoints (same fire intensity) used in the *post hoc* experiment that varied in egress path distance (**left**: closer; **right**: farther). The viewpoints were selected to display the exit to the room (far corner) and were positioned the same distance from the fire source.

An additional 80 participants completed the *post hoc* experiment (same recruitment procedure as the main experiment). To focus on path distance, one condition was selected: living room, ultra growth rate, with a smoke opacity of 5%. Two new viewpoints were selected, both 2 m from the fire, but one viewpoint with a farther path to the entrance that required walking around the fire (path distance to door; closer = 6.26 m, farther = 7.34 m; see Figure 4). To create a clear path of egress, the sofa positioned closest to the new viewpoint locations was removed from the rendered videos. All other aspects of the experiment remained the same.

Using a linear mixed model with main and interaction effects of path distance and intensity (random intercept for participant), no significant effects were observed for path distance: main effect, *F*(1.00, 81.00) = 0.00, *p* = 0.963, Cohen’s *f* = 0.01, interaction effect, *F*(1.00, 891.00) = 1.35, *p* = 0.245, Cohen’s *f* = 0.04 (see Supplementary materials for statistics of each effect). A significant effect was observed for intensity, which followed a similar pattern to the main experiment, higher risk ratings for more intense fires, *F*(1.00, 891.00) = 353.09, *p* < 0.001, Cohen’s *f* = 0.63. These results suggested that path distance did not have a significant impact on risk ratings.

## Discussion

The goals of the present study were to develop a video library of fire simulations and evaluate their use for investigating the perceived risk of developing room fires in scenario-based experiments. To do so, numerical simulations were used to render videos that visualized realistically behaving fires that developed at various growth rates in different rooms, with various smoke opacity, and from different viewpoints. We hypothesized that, if the videos were able to elicit perceptions of fire emergencies, then perceived risk ratings provided by participants would vary with these simulation characteristics. The behavioral results indicated that indeed both fire and environmental characteristics influenced participant ratings. This suggested that the simulation videos were successful in eliciting perceptions of potential harm regarding developing fires. We further suggest that this provides evidence that the videos can be used by researchers to investigate human perceptions of developing fires in scenario-based research.

### Impact of fire characteristics on perceived risk

The flame and smoke characteristics influenced the perceived risk of developing room fires. Higher risk ratings were generally observed with more intense (i.e., larger flames), more opaque smoke, and faster growing fires (see Supplementary materials for an analysis of a visibility indicator that corroborated the effect of smoke opacity). The role of growth rate and smoke opacity influenced the effect of fire intensity: intensity had less of an impact on perceived risk for slower growing and more opaque smoke fires. We posit that the interaction between fire intensity, growth, and smoke was likely due to the rate of smoke accumulation across the different conditions. The simulated fires shared the same reaction and combustion properties, including smoke production. Slower growing fires take more time to reach specific HRR intensities and thus have corresponding greater amounts of smoke produced when an HRR is reached compared to faster growing fires. A greater accumulation of smoke within the simulation environment was visually rendered as more opaque, obscuring flame visual cues. With the occlusion of visual flame cues at greater intensities and less transparent smoke, participants may have had less visual information to distinguish between the intensity of the fire. Indeed, for slower growing fires with less transparent smoke, risk ratings were similar for greater fire intensities. This indicates that the cues of growth rate and intensity may be less important when perceiving the risk of fires when visible flames are occluded, as was the case when thicker smoke has accumulated in the building.

The intersection of fire characteristics in relation to perceived risk extends prior HBIF research. Indeed, when focusing on visual characteristics of fires, past research has observed that the presence of smoke, growth rate, and intensity can each independently serve as indicators of risk (Kobes et al., 2010; Qin and Gao, 2019). The present study suggests that the impact of these characteristics on perceptions of risk are relative to each other, with the level of one influencing the effect of others. This is in line with predictions that several fire characteristics modulate the perception of risk (Kinateder et al., 2015). The present study supports this view indicating that that the impact of one category of visual characteristics of fire on risk perception is dependent on other visual fire characteristics. However, as with prior studies that used hypothetical scenarios to examine human behavior in fire, participants were not exposed to personal harm as they made perceived risk ratings in the present study. How the impact of fire characteristics on risk perception when there is an imminent danger to personal health and property compares to the present study remains to be examined. Research that has investigated layperson perceptions of disaster threats, a category which includes fires, has provided evidence that risk perception is influenced by objective hazard information and an affective reaction (Peters and Slovic, 1996). This is in line with the conceptualization of risk perception during fire evacuations that includes a subjective component, which goes beyond rational probabilities of encountering harm (Kinateder et al., 2015). With the present study focusing on the judged potential harm of a simulated fire, we argue that participant ratings were likely more heavily influenced by perceptual information, such as fire characteristics, than an affective reaction. That the effects of fire characteristics in the present study aligned with those that have been examined in prior research indicates that said characteristics are, at minimum, available for occupants to base their perceived risk when faced with a real fire.

Research that has used media to investigate emotion responses provides some predictions as to how a greater affective response may influence the impact of perceptual information on fire risk perception. In particular, the intensity of emotion responses may be lower with media-based stimuli of emotionally salient objects compared to encountering them in real-life (Lang et al., 1993; Berretz et al., 2023). Indeed, when comparing computer-generated animations of fear-related animals, which included body movement, stronger self-report ratings and physiological responses were observed, as compared to analogous real-life photos (Courtney et al., 2010). In addition, when investigating emotion responses to disgust-inducing objects, a similar pattern in self-report arousal and valence ratings were observed between photos and real-life objects with regard to neutral objects, however the intensity of the ratings was stronger for real-life objects (Berretz et al., 2023). We hypothesize that, if a similar effect of naturalism on emotion responses holds, the affective reaction to real-life fire may increase the intensity of the perceived risk of the fire, but that the overall pattern would be similar to the results of the present study. In addition to explicit behavioral responses, emotion research has used implicit psychophysiological and neuroimaging measures to investigate responses to stimuli (e.g., Cuthbert et al., 2000). The present study was limited to behavioral responses; future research can investigate whether similar patterns of fire and environmental factors are present with psychophysiological and neuroimaging measures. The presence of corroborating results across different levels of naturalism and research measures would provide additional evidence that video simulations of fires can be used to study human behaviors and perceptions of fire.

### Perceived risk across environmental factors

The type of room and location within it influenced the perceived risk ratings provided for fire videos. Videos that displayed viewpoints within a bedroom and living room and closer to the fire were rated as more dangerous. In line with theories of grounded cognition (Barsalou, 2008), the effect of room type may have been due to participants applying contextual knowledge when viewing the videos and generating their responses. Indeed, perceiving a fire within a bedroom as posing more risk compared to within a kitchen environment aligns with fire incidents being more likely to occur in kitchens than bedrooms (Thomas and Brennan, 2003; Spearpoint and Hopkin, 2020). Specifically, perceiving fires as posing greater risk in bedrooms could be due to cognitive biases, where heuristics involved in decision-making can contribute to irrational judgments to be made (Ehrlinger et al., 2016). Different cognitive biases have been proposed to influence aspects of human responses to fire, from the selective focus on hazard cues through comprehension of the information in the environment (Kinsey et al., 2018). In the present study, the conceptual knowledge about the typical activities and items found within a type of room could have contributed to cognitive biases influencing risk ratings. For example, optimistic bias (tendency to overestimate favorable outcomes) and normalcy bias (tendency to overlook abnormalities as being typical) have been proposed to contribute to cooking-related fire incidents (Murata et al., 2015). A potential account for lower perceived risk for kitchen fires in the present study is that cognitive biases minimized the judged danger since fire is used for cooking-related activities within this type of room. However, the specific cognitive biases that may have contributed to environmental differences in risk ratings remain to be determined. Nonetheless, that risk ratings varied by the type of room provides support for the use of fire simulation videos to investigate the impact of environmental factors on human perceptions of developing fires.

The virtual distance of viewpoints from the simulated fire affected risk ratings in the present study. This extends prior findings that spatial proximity to fires is an environmental factor that can influence human responses to fires (Kobes et al., 2010). We used a *post hoc* experiment to compare two competing hypotheses for the effect in the present study. The results aligned with the physical size hypothesis, that when fire cues appeared physically larger within the video frames, with viewpoints closer to the fire, participants viewed the fire as posing greater risk. However, mental imagery may still have contributed to the distance effect. In real-world situations, standing closer to a fire does indeed increase the visual extent of fire characteristics within a person’s field of view as well as exposure to combustion products in other sensory modalities including somatosensory (heat) and olfactory (smoke smell). In line with embodied cognition (Wilson, 2002), participants may have engaged in mental simulations that included some of these other modalities. If so, the intensity of these mental simulations would have varied with viewpoint distance, potentially impacting risk ratings. A path to investigate the distance effect from the embodiment perspective in future research is to examine whether there is evidence of multimodal simulations occurring when participants are presented with fire cues within a single modality, such as visual.

Visual immersion within a room may contribute to the impact of the relative location of observer to a fire with regard to risk perception. The present study was limited in that viewpoints were rendered on two-dimensional (2D) device screens. When considering the spectrum of visually immersive environments, 2D screens are lower in immersion as compared to a stereoscopic virtual reality system (Kinateder et al., 2014). Prior research has indicated that greater visual immersion within a virtual environment can increase the intensity of emotion rating responses (Estupiñán et al., 2014) and influence fire evacuation behavior (Davis et al., 2023). Future research should investigate whether the spatial placement of observers from a fire when viewed within a three dimensional (3D) virtual reality system influences risk perception. However, it is important to note that the format of the videos in the library used in the present study are not well-suited for virtual reality research; a better suited format would be 360-degree videos, similar to what has been used in emotion perception research (Somarathna et al., 2023). Based on the present study, we hypothesize that the effect of shorter viewpoint distances on greater perceived risk would be stronger within virtual reality systems.

### Validation of simulated fire video library

The modulation of risk ratings when varying fire and environmental factors indicates that fire simulation videos can be used to investigate human perceptions of building fires in scenario-based experiments. A goal of the present study was to develop a library of videos that depicted room fires with several characteristics systematically varied across numerical simulations. Ratings of perceived risk were collected by presenting snippets of these videos to determine whether behavioral performance aligned with effects reported in prior human behavior in fire research. Indeed, the present study observed the following effects reported or hypothesized by prior research: fire intensity, smoke opacity, fire growth rate, room type, and viewpoint location. The presence of these hypothesized effects provides evidence that the simulation library can be used to investigate some aspects of human perceptions of fire. Of strategic importance of the video library to HBIF research is the ability to investigate interactions between fire and environmental factors as they relate to perceptions of building fires. Numerical simulation software, such as FDS, allows for these factors to be systematically manipulated, which can be problematic to implement using real building fires due to cost and safety concerns (Arias et al., 2021). The results of the present study suggest that, even when limited to visual stimuli, renderings of simulated fires can elicit effects of situational factors with perceived risk ratings. As such, the present study provides evidence that videos of simulated fires can be utilized to examine perceptions of the potential harm posed by building fires. However, it is important to emphasize that the ecological validity of simulated fires in scenario-based research is limited and further research is required to investigate how risk perception in such experiments, including the present study, varies from real-life fire events.

### Limitations and future directions

A fundamental limitation of the present study is the lack of physical and psychological harm posed to participants. Similar to prior studies (Lovreglio et al., 2015; Hulse et al., 2020), at no point were participants at risk of experiencing the dangers of a real building fire. This is similar to efforts using immersive simulations in human behavior in fire research as well – even when using multimodal stimuli (heat lamps, smoke odorant), participants are still not at risk of harm from a simulated fire (Shaw et al., 2019). Like prior research that used simulated fires, caution is warranted in applying the results of the present study and behaviors observed with video library simulations to occupant actions during real fire incidents. Additional research is required to identify the extent to which the effects observed in the present study transfer to situations that are closer to real-world incidents. Previous studies with immersive environments provide an approach for eliciting “behavioral realism” in fire safety science (Arias et al., 2021). For example, Shaw et al. (2019) presented characteristics of real fires to participants as they completed fire scenarios in immersive multimodal environments. Taking a similar approach with the present study, the output of numerical simulations can be used to present multimodal sensory stimuli as situational factors are manipulated; the observation of similar patterns in behavior within these immersive simulations would provide greater evidence that said effects may also be observed within real-world incidents.

The level of visual realism of the simulated fires as rendered in the video libraries was limited by the capabilities of commercially available and open-source software. As discussed previously, the use of FDS to simulate fires was intentional as it is used to model the dynamics of fires and implemented in performance-based design approaches for building life safety planning. The visualization capabilities of FDS and Smokeview are primarily intended to animate the output of simulations as well as communicate the results to stakeholders (Forney, 2023). This is distinct from the goals of visual effects software used to render photo-realistic fires in video games and motion films (e.g., EmberGen, JangaFX), media formats that the general public may be familiar with. As such, the videos are limited in providing photo-realistic fires. However, fire simulations have been successfully used to study aspects of human behavior in response to fire, including the present study. We recommend that researchers consider including additional context when using the video library in their studies. For example, providing scenarios that describe what object is burning (with the limitations that it takes the form of the cubic structure used in the simulations) and information about the building in which the room is located could aid in providing greater context for interpreting the situation. The present study lacked such contextual descriptions and presented simulated fire videos in an artificial approach, such as having participants repeatedly rate the perceived risk of fires within the same room. These deviations from realism were intended to maintain consistency across conditions. However, these mark opportunities for future research to examine whether providing descriptions in specific situations and using experiment procedures that align more closely with realistic sequences of events impacts how fire characteristics influence the perceived risk of a simulated fire.

The online task used in the present study to evaluate the perceived risk of simulated fires poses limitations on the applicability of the findings. An online recruitment method was used to collect behavioral ratings from a large sample of participants. However, in doing so, the approach entailed less control over the environment in which the experiment was completed as well as the types of data that were collected. Participants were able to use any internet-connected device to complete the study, including desktop computer and mobile devices. Said devices vary in multiple ways, including the size and brightness of the screen that the videos were displayed. Although we were not able to collect the physical dimensions of the devices used to complete the study, the tasks recorded the initial screen resolution of the browser window when participants initially accessed the study and whether it was a mobile device. An exploratory comparison did not yield statistically significant differences in ratings across these properties (see Supplementary materials). Although mobile devices are generally smaller in physical size than desktop devices, it remains to be investigated whether physical size affects the perceived risk of video fires. Based on the *post hoc* experiment, we hypothesize that the relative size to the screen, rather than the physical real-world screen size, of the fires impacts perceived risk ratings. Subsequent research can investigate this prediction by systematically manipulating the physical size of smaller versus larger intensity fires as displayed on screens. In comparison to prior emotion research, the present study was limited in collecting explicit behavioral ratings from participants. Past studies examining emotional responses to media stimuli have collected both behavioral and psychophysiological responses (Lang et al., 1993; Cuthbert et al., 2000; Berretz et al., 2023). In doing so, patterns in multimodal responses to emotion have been observed, providing physiological and neuroimaging evidence that corroborates patterns observed in behavioral responses. Future research that takes a similar approach with simulated fire videos can examine whether common patterns in response to the manipulation of fire characteristics are observed with psychophysiological and behavioral measures. The presence of corresponding effects across psychophysiological and behavioral measures would provide additional evidence that simulated videos can be used to examine the perceived risk of building fires in laboratory experiments.

There are multiple directions that future research can pursue with the library of fire videos. Prior HBIF research provides several observations that can be systematically examined, including the impact of having other persons within the building on the risk posed by a fire (social cues) as well as fire and smoke alerting devices (Kinsey et al., 2018). Identifying how fire characteristics and environmental factors interact with these variables can provide a broader understanding of the variables that shape human perceptions of fire risk. As with prior research (Bonny and Milke, 2023), individual variation was observed with fire perception: in the present study some participants perceived fires as less risky than other participants. Understanding how these individual differences are connected to personal characteristics is another avenue for future research. The library of fire simulation videos described in the present research provides a resource for fire and social science researchers to address these future directions in HBIF.

## Data Availability

The datasets presented in this study can be found in online repositories. The names of the repository/repositories and accession number(s) can be found below. Study datasets can be found on OSF (https://doi.org/10.17605/OSF.IO/QA4SU). Validation video files and simulation materials can be found on FigShare: https://doi.org/10.25452/figshare.plus.25888315, https://doi.org/10.25452/figshare.plus.25888198, and https://doi.org/10.25452/figshare.plus.25888147.
