# Supplementary material for: Simulated fire video collection for advancing understanding of human behavior in building fires
Source: Front Psychol. 2024 Aug 26;15:1438020. doi: 10.3389/fpsyg.2024.1438020 (PMC11382246; doi:10.3389/fpsyg.2024.1438020)
Supplement: Supplementary file 1 [file Data_Sheet_1.docx]

# Simulated Fire Video Collection for Advancing Understanding of Human Behavior in Building Fires

# Supplementary Materials

Justin W. Bonny ^1^, Zishanul Hussain ^2^, Micah D. Russell ^1^, Arnaud Trouvé ^2^, James A. Milke ^2^

^1^ Department of Psychology, Morgan State University

^2^ Department of Fire Protection Engineering, University of Maryland, College Park

# Fire Simulation Parameters

The computation model parameters for the Fire Dynamics Simulator (FDS) simulations were selected using prior specifications (Hurley et al., 2015; McGrattan et al., 2021). The combustion parameters were set according to those of polyurethane (Table S1). All FDS input files can be access using the following online repository (Bonny, 2024a). The “.fds” input files can be opened using FDS and the “.psm” files can be opened via PyroSim. Note that the image files included in the repository have been recolored and will not match the video files when viewed via Smokeview (Forney, 2023).

Table S1

*Combustion properties for polyurethane used in the fire simulations.*

| Property ^a^ | Value | Measurement Units |
| --- | --- | --- |
| C | 1 | - |
| H | 1.7 | - |
| O | 0.3 | - |
| N | 0.08 | - |
| Soot Yield | 0.198 | g/g |
| CO Yield | 0.042 | g/g |

^a^ C = carbon; H = hydrogen; O = oxygen; N = nitrogen; CO = carbon monoxide

# Simulation Output and Rendering

## Computational Output

The present study utilized the heat release rate (HRR) modeled in FDS to select the portion of each simulation to render as part of the validation efforts. The HRR output for each simulation can be accessed via an online repository (Bonny, 2024a). The file provides the modeled HRR at each timeframe of the simulation. Additional output files were generated using FDS devices, however they were not the focus of the present study.

The complete simulation output files can be accessed by contacting the corresponding author. Although we anticipate that the video library will be sufficient for researchers to use in a variety of human behavior in fire research projects, the output files can be used to render immersive environments. Specifically, Smokeview and PyroSim can render simulation outputs as 360-degree images and videos that can be viewed within virtual reality systems. In addition, PyroSim has virtual reality functionality that allows users to navigate the simulated environment while fires are visually rendered. We recommend that those interested in using the output files in this and similar extensions of the present study review the technical documentation for each software application prior to obtaining the simulation files (the files are substantial in disk size, greater than 200 GB each, and require a high-speed internet connection to download).

## Visually Rendered Smoke Opacity

The video libraries were based on a material and combustion specification (polyurethane) that produced smoke that was sootier (i.e., produced more particulate material) than other types (e.g., pine wood). As a result, when using the default parameters for visually rendering smoke viewpoints closer to the fire were rapidly occluded, only displaying dark gray color for the entire frame. To adjust for this, and investigate the impact of varying smoke transparency, the opacity settings in PyroSim were adjusted to be lower than the default setting of 100% (0% = smoke particulate was invisible; 100% = smoke particulate was opaque). As such, although the numerical model of the simulations aligned with the polyurethane specifications, the visual rendering of the smoke did not align with the particulate generated by a polyurethane combustion. For the intentions of the video library as a means for manipulating fire properties to investigate the impact on human perceptions we believe this was acceptable. However, the videos do not display realistic smoke generated from polyurethane combustion and should not be used to determine how humans respond to polyurethane-specific fires.

# Validation Materials and Supplementary Results

## Validation Video Files

All validation files are available via an online repository (Bonny, 2024b).

## Supplementary Results

The effects for each term in the accepted reduced model are presented in Table S2. The post hoc contrasts for room type are presented in Table S3.

Table S2

*Linear mixed model effects predicting risk ratings.*

| Effect | Sum Sq | Mean Sq | Num. DF | Den. DF | F value | P value | Cohen’s *f* |
| --- | --- | --- | --- | --- | --- | --- | --- |
| Growth rate | 23.42 | 23.42 | 1 | 2158 | 14.79 | 0.0001235 | 0.08279 |
| Intensity | 10327 | 10327 | 1 | 23738 | 6524 | 0 | 0.5242 |
| Opacity | 2.367 | 2.367 | 1 | 2158 | 1.495 | 0.2215 | 0.02632 |
| Viewpoint Distance | 16.13 | 16.13 | 1 | 2158 | 10.19 | 0.001435 | 0.06871 |
| Room Type | 35.05 | 11.68 | 3 | 2158 | 7.381 | 6.413e-05 | 0.1013 |
| Growth rate × Intensity | 405.5 | 405.5 | 1 | 23738 | 256.2 | 2.345e-57 | 0.1039 |
| Growth rate × Opacity | 6.11 | 6.11 | 1 | 2158 | 3.86 | 0.04958 | 0.04229 |
| Intensity × Opacity | 1714 | 1714 | 1 | 23738 | 1083 | 2.65e-232 | 0.2136 |
| Growth Rate × Intensity × Opacity | 333.1 | 333.1 | 1 | 23738 | 210.4 | 1.777e-47 | 0.09415 |

Table S3

*Post hoc contrasts comparing ratings across room types (p-values adjusted using Sidak corrections).*

| Contrast | Estimate | SE | Z ratio | Adj. p-value |
| --- | --- | --- | --- | --- |
| bedroom - kitchen | 0.3858 | 0.09344 | 4.129 | 0.0002187 |
| bedroom – living room | 0.1014 | 0.09159 | 1.107 | 0.8465 |
| bedroom - office | 0.3072 | 0.09159 | 3.354 | 0.004767 |
| kitchen – living room | -0.2844 | 0.09396 | -3.027 | 0.01473 |
| kitchen - office | -0.0786 | 0.09397 | -0.8365 | 0.9547 |
| Living room - office | 0.2058 | 0.09212 | 2.234 | 0.1434 |

## Browser Characteristics Results

Using the jsPsych 'browser-check' plugin, the initial characteristics of the browsers participants used to access the study were recorded; this included the size of the browser window (in pixels; the diagonal was calculated using browser width and height) and whether the browser was running on a mobile device. Note that these were initial settings - if the participant resized the browser after initially accessing the study, or if the participant was emulating a device (e.g., mobile device emulating a desktop computer), these characteristics would be inaccurate. With these limitations considered, an exploratory analysis investigated whether the browser diagonal size (scaled and centered) and mobile device type influenced risk ratings in conjunction with viewpoint distance in the main experiment. Across conditions, a total of 1637 participants were detected as using a browser on a desktop device (screen diagonal: *M* = 1795.56, *SD* = 382.30, *Min* = 688.77, *Max* = 3682.77) and 521 on a mobile device (screen diagonal: *M* = 812.26, *SD* = 118.52, *Min* = 517.24, *Max* = 1391.71). However, no significant main effects of browser characteristics nor interactions with viewpoint distance were observed (*p*s > .1; see Table S4).

Table S4

*Linear mixed model browser characteristic and viewpoint distance effects predicting risk ratings.*

| Effect | Sum Sq | Mean Sq | Num. DF | Den. DF | F value | P value | Cohen’s *f* |
| --- | --- | --- | --- | --- | --- | --- | --- |
| Screen Diagonal | 2.9 | 2.9 | 1 | 2158 | 1.4 | 0.24 | 0.025 |
| Mobile Device | 2.2 | 2.2 | 1 | 2158 | 1.1 | 0.3 | 0.022 |
| Viewpoint Distance | 0.018 | 0.018 | 1 | 2158 | 0.0083 | 0.93 | 0.002 |
| Screen Diagonal × Mobile Device | 3.5 | 3.5 | 1 | 2158 | 1.7 | 0.2 | 0.028 |
| Screen Diagonal × Viewpoint Distance | 0.68 | 0.68 | 1 | 2158 | 0.32 | 0.57 | 0.012 |
| Mobile Device × Viewpoint Distance | 0.42 | 0.42 | 1 | 2158 | 0.2 | 0.66 | 0.0095 |
| Screen Diagonal × Mobile Device × Viewpoint Distance | 0.32 | 0.32 | 1 | 2158 | 0.15 | 0.7 | 0.0084 |

## Modified Visibility Analysis

The numerical simulation software (FDS) can estimate the visible distance at which an occupant can see through particulate generated by the fire within a space. In the present study, this measure of visibility was recorded at each viewpoint for all simulations (maximum visibility is 30 m by default). However, the opacity of the visually rendered smoke was manipulated post simulation, when rendering videos of the simulation via PyroSim. The FDS measure of visibility does not account for this post simulation processing. This means that the smoke rendered within the fire videos does not reflect the visibility metric calculated by FDS during the simulation. However, an exploratory analysis was run to evaluate whether a modified estimate of visibility aligned with the analysis of smoke opacity included in the main text.

To estimate a metric of visible distance that incorporates the impact of manipulating opacity, the FDS visibility measures were transformed by the percent of opacity applied when rendering the video, with the maximum clamped to the 30 m default (e.g., 1 m visibility estimated by FDS ÷ [10% opacity] = 10.0 m; 1 m visibility estimated by FDS ÷ [5% opacity] = 20.0 m). For each video clip, we calculated the mean visible distance from the start through end time of video within the simulation. Note that visible distance and smoke opacity are inversely related: greater opaqueness (higher smoke opacity) is associated with lower visibility.

Next, we used a linear mixed model that included a main effect of mean visible distance (scaled and centered) to predict response ratings. The main effect of visible distance was significant, and aligned with the results described in the manuscript: a lower visible distance was associated with greater risk ratings, *t*(24905.87) = -32.81, *p* < .001. This provided corroborating evidence that metrics indicating the level of visual occlusion as a result of developing fires were associated with perceived risk.

It is important to note that this estimated metric does not represent actual visibility. This approach should not be used to calculate the actual distance through which occupants can see through a simulated fire. An alternate approach to adjusting smoke opacity in PyroSim post-simulation would have been to modify the 'soot yield' parameter of chemical reactions in the FDS pre-simulation. However, we did not do so as this would have altered the chemical reaction, leading to inconsistencies across simulations.

# Post Hoc Experiment Materials and Supplementary Results

## Post Hoc Video Files

All validation files are available via an online repository (Bonny, 2024b).

## Post Hoc Experiment Supplementary Results

The effects for each term in the accepted reduced model are presented in Table S5.

Table S5

*Linear mixed model effects predicting risk ratings during post hoc experiment.*

| Effect | Sum Sq | Mean Sq | Num. DF | Den. DF | F value | P value | Cohen’s *f* |
| --- | --- | --- | --- | --- | --- | --- | --- |
| Path Distance | 0.003505 | 0.003505 | 1 | 81 | 0.002143 | 0.9632 | 0.005144 |
| Intensity | 577.3 | 577.3 | 1 | 891 | 353.1 | 1.297e-66 | 0.6295 |
| Path Distance × Intensity | 2.215 | 2.215 | 1 | 891 | 1.355 | 0.2448 | 0.03899 |

# References

Bonny, J. W. (2024a, August). *Input and Numerical Output of Simulated Fire Video Library*. Figshare+. https://doi.org/10.25452/figshare.plus.25888198

Bonny, J. W. (2024b, August). *Video Clips Used to Validate Simulated Fire Video Library*. Figshare+. https://doi.org/10.25452/figshare.plus.25888315

Forney, G. P. (2023). *Technical Reference Guide* (NIST Special Publication 1017-2; Smokeview, A Tool for Visualizing Fire Dynamics Simulation Data). National Institute of Standards and Technology.

Hurley, M. J., Gottuk, D. T., Jr, J. R. H., Harada, K., Kuligowski, E. D., Puchovsky, M., Torero, J. ́ L., Jr, J. M. W., & WIECZOREK, C. J. (2015). *SFPE Handbook of Fire Protection Engineering*. Springer.

McGrattan, K., Hostikka, S., McDermott, R., Floyd, J., Weinschenk, C., & Overholt, K. (2021). *Fire dynamics simulator user’s guide* (SP 1019; p. 386). National Institute of Standards and Technology.
